# Immunohistochemical expression of epidermal growth factor receptor (EGFR) in South Asian head and neck squamous cell carcinoma: association with various risk factors and clinico-pathologic and prognostic parameters

**DOI:** 10.1186/s12957-018-1425-3

**Published:** 2018-06-28

**Authors:** Atif Ali hashmi, Zubaida Fida Hussain, Saher Aijaz, Muhammad Irfan, Erum Yousuf Khan, Samreen Naz, Naveen Faridi, Amir Khan, Muhammad Muzzammil Edhi

**Affiliations:** 1 Liaquat National Hospital and Medical College, Karachi, Pakistan; 2 Shaheed Zulfiqar Ali Institute of Science and Technology, Karachi, Pakistan; 3 Kandahar University, Kandahar, Afghanistan; 4 Brown University, Providence, RI USA

**Keywords:** Epidermal growth factor receptor, Head and neck squamous cell carcinoma, Oropharyngeal squamous cell carcinoma, Areca nut, Gutka

## Abstract

**Background:**

In this study, we intend to determine the immunohistochemical expression of EGFR in cases of head and neck squamous cell carcinoma and its association with prognostic clinico-pathologic features.

**Methods:**

A total of 115 cases of head and neck squamous cell carcinoma treated at Liaquat National Hospital, Karachi, Pakistan, were included in the study. Clinico-pathologic features, risk factors, and recurrence status of cases were evaluated, and EGFR immunohistochemistry was performed.

**Results:**

In our study, 52 cases (45.2%) of head and neck SCC were positive and 10 cases (8.7%) were focal positive for EGFR expression, while 53 cases (46.1%) were negative for EGFR expression. High EGFR expression (> 70%) was noted in 6.1% (7 cases), while 12.2% (14 cases) and 26.1% (30 cases) revealed 51–70% and 11–50% EGFR expression respectively. On the basis of intensity, strong EGFR expression was noted in 13.9% (16 cases) while 16.5% (19 cases) and 23.5% (27 cases) revealed intermediate and weak EGFR expression respectively. Significant association of EGFR expression was noted with tumor stage and disease-free survival.

**Conclusion:**

We found a significant association of EGFR expression with tumor stage and disease-free survivals, which are the most important prognostic factors in head and neck squamous cell carcinoma; therefore, EGFR expression can help as a prognostic biomarker in head and neck squamous cell carcinoma. On the other hand, we suggest that molecular studies should be performed in squamous cell carcinoma of head and neck in our setup to identify patients that can avail response from anti-EGFR therapy.

## Background

With a global incidence of 500,000 per annum, squamous cell carcinoma has been reported to be the most prevalent cancer of the oral cavity [1]. In South Asia, head and neck cancers are the third leading cause of cancer-related morbidity and mortality [2–4]. Approximately 90 to 95% of oral squamous cell carcinoma (SCC) shows varied degrees of epithelial dysplasia [5]. Molecular carcinogenesis of head and neck squamous cell carcinoma (HNSCC) is attributed to several cytogenetic alterations in oncogenes and receptors for growth factors including p53, p27, p16, cyclin D1, and epidermal growth factor receptor (EGFR) [6, 7]. Cellular growth differentiation and proliferation rely on the growth factor-induced stimulation of the cellular processes. EGFR plays a substantial role in differentiation and proliferation of the mammalian cells [8]. Expression of EGFR in a number of epithelial cell tumors in humans has been well documented, and 80% of squamous cell carcinomas are marked by over-expression of EGFR, resulting in proliferation and differentiation of keratinocytes [9–11]. Squamous cell carcinomas of the head and neck exhibit a varying degree of behavior apropos of cellular growth rate, differentiation, and metastasis. In our setup, areca nut/gutka chewing has been asserted as a major risk factor attributable to the development of leukoplakia and subsequent SCC of oral cavity. In the era of personalized medicine, it is largely accepted that cancer therapy protocols should be devised in the light of tumor characteristics of loco-regional population.

Previous studies revealed that immunohistochemical over-expression of EGFR correlates with poor prognosis [12]; moreover, EGFR over-expression is considered as an attractive target for anti-EGFR therapy in various tumors. Therefore, in the present study, we studied the association of EGFR over-expression with unfavorable prognostic features including advanced tumor grade, tumor size, nodal metastasis, and recurrence status in our population.

## Methods

This was a retrospective study conducted in the Department of Histopathology, Liaquat National Hospital from January 2008 till December 2013. The study duration was 7 years. One hundred fifteen cases of biopsy-proven squamous cell carcinoma were included in the study. All patients underwent radical excisions of cancer along with neck dissection from level I to level V. The study was approved from research and ethical review committee of Liaquat National Hospital. Slides of all cases were retrieved from records of pathology department and reviewed by two surgical pathologists to determine tumor characteristics including histological type, grade, T-stage, N-stage, and perineural and lymphovascular invasion. Representative tissue blocks of all cases were selected for immunohistochemistry.

Clinical records of 45 patients were available. Many patients lost to follow-up or history of risk factors were not mentioned in patient records. Clinical records of these patients were reviewed from institutional records to evaluate patients’ age, smoking, alcohol and gutka/pan use history, history of radiation and chemotherapy, and recurrence status.

### Immunohistochemistry

**Fig. 1:**
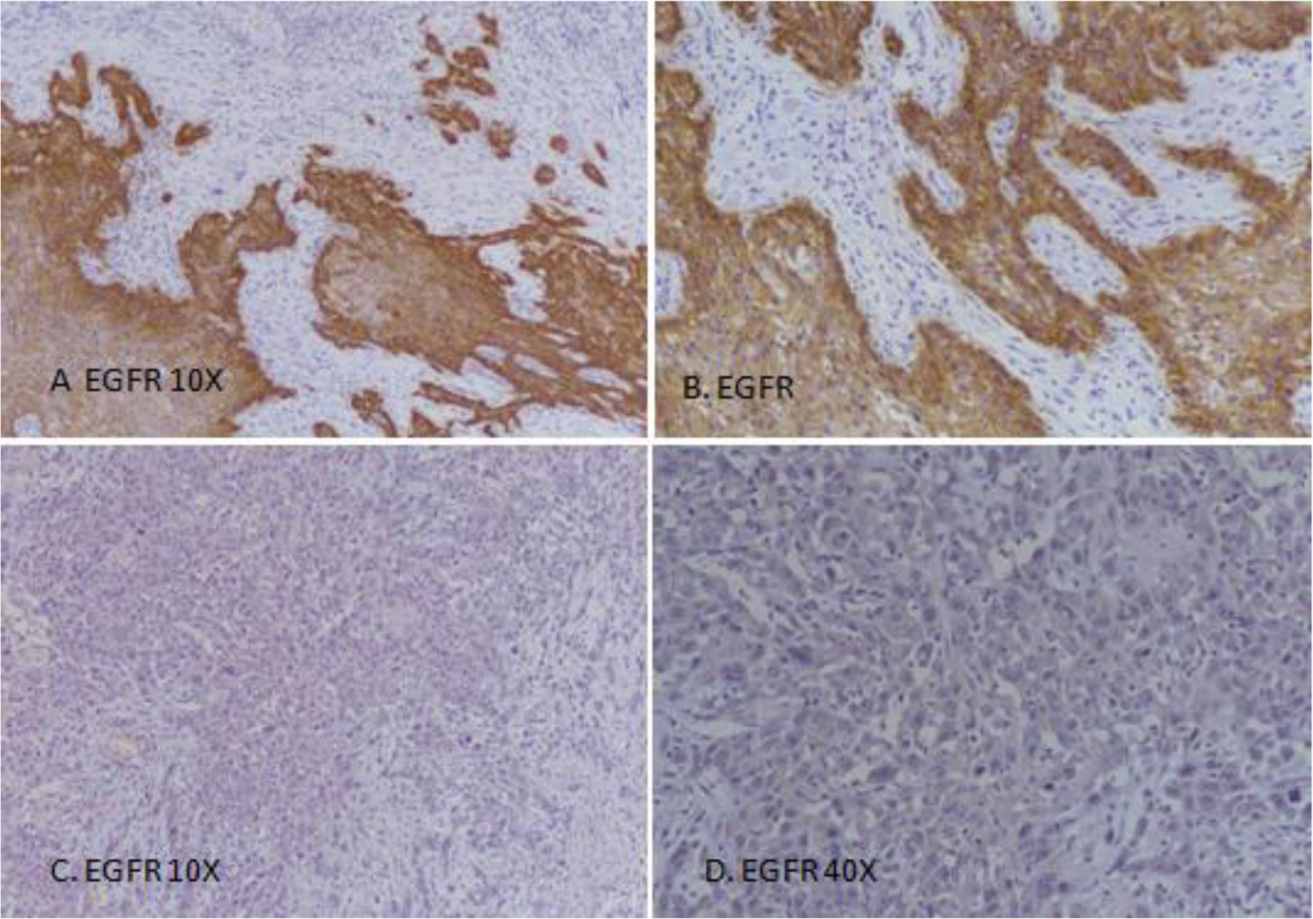
EGFR expression in head and neck squamous cell carcinoma. **a**, **b** Positive EGFR expression 3+, > 70% cells; **c**, **d** Negative EGFR expression

EGFR immunohistochemistry was done using DAKO EnVision method and DAKO Monoclonal Mouse Anti-human Epidermal growth factor Receptor (EGFR), clone H11 as per manufacturer recommendations. Both membranous and cytoplasmic staining for EGFR was evaluated. Intensity of staining was assorted into no staining (0), weak (1+), intermediate (2+), and strong (3+) while percentage of positively stained cells was counted. Intermediate to strong staining in > 10% of tumor cells was considered positive while weak to intermediate staining in < 10% of cancer cells was taken as focal positive (Fig. 1). Moreover, EGFR immunostaining was also categorized according to percentage of staining cells into different groups as shown in Table 2.

### Follow-up and recurrence

Hospital file records were evaluated to determine recurrence status of the patients. Disease-free survival was defined as time from surgical resection till first recurrence, patient’s death, or last medical follow-up. Overall survival was labeled as time from primary treatment till death or last follow-up. None of the patients received anti-EGFR therapy.

### Statistical analysis

Statistical package for social sciences (SPSS 21) was adopted for data analysis. Mean and standard deviation were evaluated for quantitative variables. Frequency and percentage were calculated for qualitative variables. Chi-square was applied to determine association. Kaplan-Meier method was used to determine survival, while significance of difference between survival curves was evaluated using log-rank ratio. *P* value of ≤ 0.05 was taken as significant.

## Results

### Clinico-pathologic features of squamous cell carcinoma of the head and neck

Table 1 shows demographic characteristics of the studied population. Mean age of the patients was 51.95 ± 12.62. Majority of patients were males (73.9%). History of addiction could only be evaluated in 44 patients out of whom 27 patients (61.4%) revealed addicted with gutka/pan. No appreciable addiction was noted with other factors like smoking or alcohol. The most common tumor location was the oral cavity (68.7%). Majority of patients were found to be at tumor stage T2 (47%). More than 2 cm tumor depth was noted in 17% of cases. Nodal metastasis was seen in 45.2% of cases, while majority of tumors were keratinizing subtype (56.5%) and of grade II (61.7%). Adjuvant radiation and chemotherapy were given in 53.3 and 51.1% of cases respectively. 51.1% of cases recurred after primary treatment.

**Table 1:** Clinico-pathologic features of squamous cell carcinoma head and neck (*n* = 115)

Characteristic	Frequency (%)
Age(years)^a^	51.95 ± 12.62
Age groups	
≤ 30 years	4 (3.5)
31–50 years	57 (49.6)
> 50 years	54 (47)
Gender	
Male	85 (73.9)
Female	30 (26.1)
History of pan (*n* = 44)	
Yes	27 (61.4)
No	17 (38.6)
History of smoking(*n* = 44)	
Yes	4 (9.1)
No	40 (90.9)
History of alcohol (*n* = 44)	
Yes	1 (2.3)
No	43 (97.7)
Location of tumor	
Oral cavity	79 (68.7)
Lip	3 (2.6)
Tongue	29 (25.2)
Soft palate	4 (3.5)
Tumor size (cm)^a^	3.21 ± 1.74
Tumor stage	
T1	31 (27)
T2	54 (47)
T3/T4	30 (26.1)
Depth of invasion (cm)^a^	1.11 ± 0.74
Depth of invasion	
< 2 cm	98 (85.2)
≥ 2 cm	17 (14.8)
Nodal stage	
No	63 (54.8)
N1	17 (14.8)
N2a	0 (0)
N2b	31 (27)
N2c	3 (2.6)
N3	1 (0.9)
Extranodal extention	
Not present	85 (73.9)
Present	30 (26.1)
Histological subtypes	
Non-keratinizing	17 (14.8)
Keratinizing	65 (56.5)
Non-keratinizing with maturation	33 (28.7)
Histologic grade	
Grade-I	31 (27)
Grade-II	71 (61.7)
Grade-III	13 (11.3)
Lymphovascular invasion	
Not present	114 (99.1)
Present	1 (0.9)
Perineural invasion	
Not present	99 (86.1)
Present	16 (13.9)
Radiation (*n* = 45)	
Yes	25 (55.6)
No	20 (44.4)
Chemotherapy (*n* = 45)	
Yes	24 (53.3)
No	21 (46.7)
Recurrence (*n* = 45)	
Yes	23 (51.1)
No	22 (48.9)

^a^Mean ± SD

### EGFR immunohistochemistry and association with clinico-pathologic parameters

**Fig. 2:**
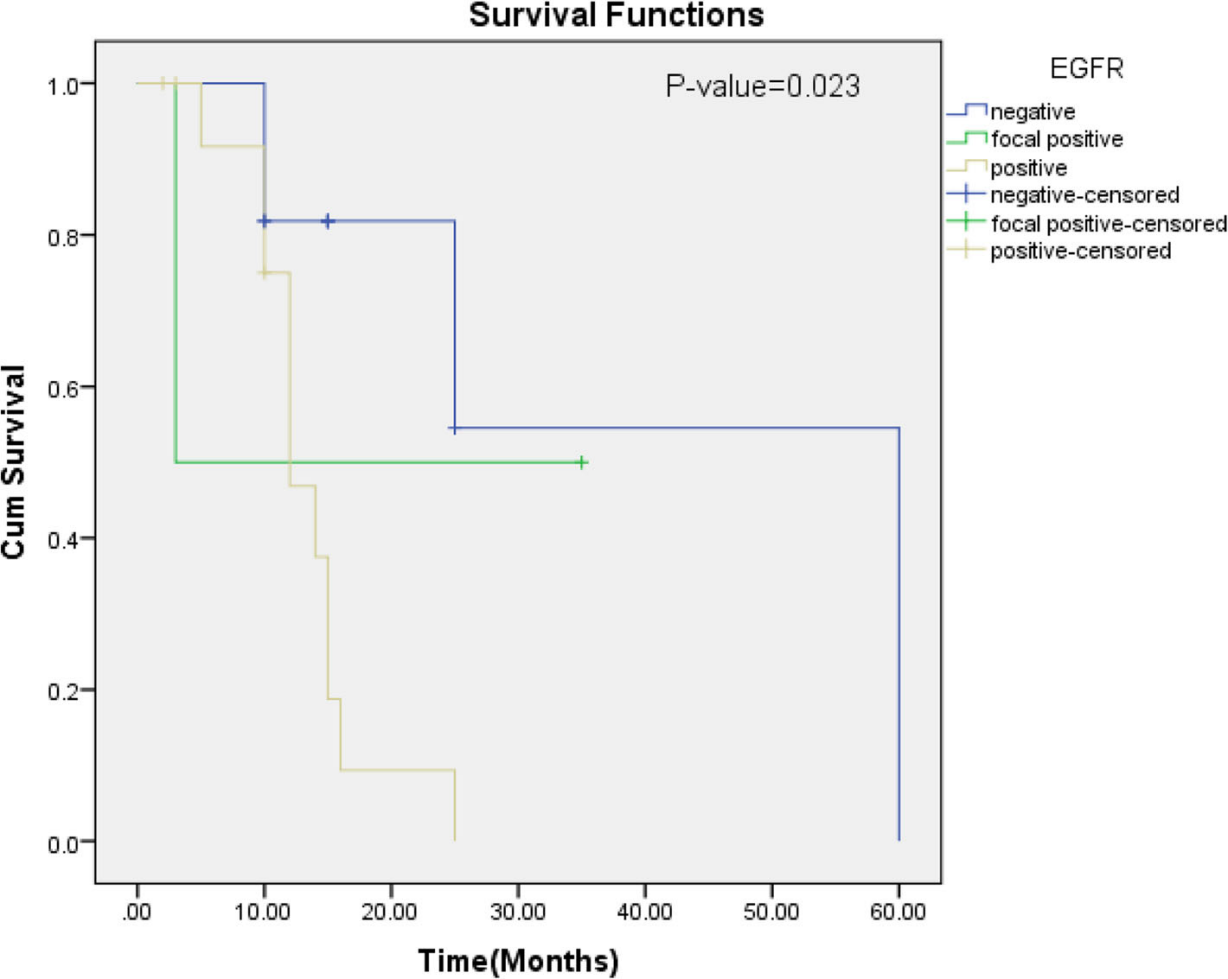
Kaplan-Meier for EGFR over-expression (disease-free survival)

In our study, 52 cases (45.2%) of head and neck SCC were positive and 10 cases (8.7%) were focal positive for EGFR expression, while 53 cases (46.1%) were negative for EGFR expression. Figure  1 shows percentage of EGFR expression in head and neck squamous cell carcinoma. High EGFR expression (> 70%) was noted in 6.1% (7 cases), while 12.2% (14 cases) and 26.1% (30 cases) revealed 51–70% and 11–50% EGFR expression respectively. On the basis of intensity, strong EGFR expression was noted in 13.9% (16 cases) while 16.5% (19 cases) and 23.5% (27 cases) revealed intermediate and weak EGFR expression respectively. Association of EGFR expression intensity and percentage revealed significant association of EGFR expression with tumor stage, while no significant association was noted with other prognostic parameters and risk factors (Tables 2 and 3). Significant association of EGFR expression was noted with recurrence status of the patients (Fig. 2).

**Table 2:** Association of EGFR expression categories (percentage) with clinico-pathologic parameters of head and neck squamous cell carcinoma

	*n* (%)					*P* value
	≤ 10% (*n* = 64)	11–50% (*n* = 30)	51–70% (*n* = 14)	> 70% (*n* = 7)	Total (*n* = 115)	
Age group						
≤ 30 years	1 (1.6)	1 (3.3)	2 (14.3)	0 (0)	4 (3.5)	0.334
31–50 years	34 (53.1)	12 (40)	7 (50)	4 (57.1)	57 (49.6)	
> 50 years	29 (45.3)	17 (56.7)	5 (35.7)	3 (42.9)	54 (47)	
Gender						
Male	45 (70.3)	24 (80)	11 (78.6)	5 (71.4)	85 (73.9)	0.793
Female	19 (29.7)	6 (20)	3 (21.4)	2 (28.6)	30 (26.1)	
History of pan (*n* = 44)						
Yes	14 (56)	7 (70)	2 (66.7)	4 (66.7)	27 (61.4)	0.917
No	11 (44)	3 (30)	1 (33.3)	2 (33.3)	17 (38.6)	
History of smoking (*n* = 44)						
Yes	4 (16)	0 (0)	0 (0)	0 (0)	4 (9.1)	0.497
No	21 (84)	10 (100)	3 (100)	6 (100)	40 (90.9)	
History of alcohol(*n* = 44)						
Yes	0 (0)	1 (10)	0 (0)	0 (0)	1 (2.3)	0.432
No	25 (100)	9 (90)	3 (100)	6 (100)	43 (97.7)	
Location of tumor						
Oral cavity	47 (73.4)	18 (60)	10 (71.4)	4 (57.1)	79 (68.7)	0.369
Lip	1 (1.6)	1 (3.3)	1 (7.1)	0 (0)	3 (2.6)	
Tongue	12 (18.8)	11 (36.7)	3 (21.4)	3 (42.9)	29 (25.2)	
Soft palate	4 (6.3)	0 (0)	0 (0)	0 (0)	4 (3.5)	
Tumor stage						
T1	14 (21.9)	13 (43.3)	2 (14.3)	2 (28.6)	31 (27)	0.013
T2	38 (59.4)	10 (33.3)	4 (28.6)	2 (28.6)	54 (47)	
T3/T4	12 (18.8)	7 (23.3)	8 (57.1)	3 (42.9)	30 (26.1)	
Depth of invasion						
< 2 cm	54 (84.4)	28 (93.3)	10 (71.4)	6 (85.7)	98 (85.2)	0.271
≥ 2 cm	10 (15.6)	2 (6.7)	4 (28.6)	1 (14.3)	17 (14.8)	
Nodal stage						
No	35 (54.7)	20 (66.7)	4 (28.6)	4 (57.1)	63 (54.8)	0.082
N1	11 (17.2)	2 (6.7)	4 (28.6)	0 (0)	17 (14.8)	
N2a	0 (0)	0 (0)	0 (0)	0 (0)	0 (0)	
N2b	17 (26.6)	6 (20)	6 (42.9)	2 (28.6)	31 (27)	
N2c	0 (0)	2 (6.7)	0 (0)	1 (14.3)	3 (2.6)	
N3	1 (1.6)	0 (0)	0 (0)	0 (0)	1 (0.9)	
Extranodal extention						
Not present	51 (79.7)	23 (76.7)	7 (50)	4 (57.1)	85 (73.9)	0.089
Present	13 (20.3)	7 (23.3)	7 (50)	3 (42.9)	30 (26.1)	
Histological subtypes						
Non-keratinizing	10 (15.6)	3 (10)	3 (21.4)	1 (14.3)	17 (14.8)	0.527
Keratinizing	37 (57.8)	20 (66.7)	5 (35.7)	3 (42.9)	65 (56.5)	
Non-keratinizing with maturation	17 (26.6)	7 (23.3)	6 (42.9)	3 (42.9)	3 (28.7)	
Histologic grade						
Grade-I	20 (31.3)	9 (30)	1 (7.1)	1 (14.3)	31 (27)	0.592
Grade-II	37 (57.8)	18 (60)	11 (78.6)	5 (71.4)	71 (61.7)	
Grade-III	7 (10.9)	3 (10)	2 (14.3)	1 (14.3)	13 (11.3)	
Lymphovascular invasion						
Not present	63 (98.4)	30 (100)	14 (100)	7 (100)	114 (99.1)	1.000
Present	1 (1.6)	0 (0)	0 (0)	0 (0)	1 (0.9)	
Perineural invasion						
Not present	57 (89.1)	26 (86.7)	12 (85.7)	4 (57.1)	99 (86.1)	0.159
Present	7 (10.9)	4 (13.3)	2 (14.3)	3 (42.9)	16 (13.9)	
Radiation (*n* = 45)						
Yes	17 (65.4)	5 (50)	0 (0)	3 (50)	25 (55.6)	0.174
No	9 (34.6)	5 (50)	3 (100)	3 (50)	20 (44.4)	
Chemotherapy(*n* = 45)						
Yes	17 (65.4)	4 (40)	0 (0)	3 (50)	24 (53.3)	0.128
No	9 (34.6)	6 (60)	3 (100)	3 (50)	21 (46.7)	

Chi-square test was applied

*P* value ≤ 0.05 was considered as significant

**Table 3:** Association of EGFR expression intensity with clinico-pathologic parameters of head and neck squamous cell carcinoma

	*n* (%)					*P* value
	No intensity (*n* = 53)	Weak (*n* = 27)	Intermediate (*n* = 19)	Strong (*n* = 16)	Total (*n* = 115)	
Age group						
≤ 30 years	1 (1.9)	0 (0)	1 (5.3)	2 (12.5)	4 (3.5)	0.447
31–50 years	27 (50.9)	12 (44.4)	10 (52.6)	8 (50)	57 (49.6)	
> 50 years	25 (47.2)	15 (55.6)	8 (42.1)	6 (37.5)	54 (47)	
Gender						
Male	37 (69.8)	21 (77.8)	16 (84.2)	11 (68.8)	85 (73.9)	0.628
Female	16 (30.2)	6 (22.2)	3 (15.9)	5 (31.3)	30 (26.1)	
History of pan (*n* = 44)						
Yes	11 (55)	6 (66.7)	5 (62.5)	5 (71.4)	27 (61.4)	0.915
No	9 (45)	3 (33.3)	3 (37.5)	2 (28.6)	17 (38.6)	
History of smoking (*n* = 44)						
Yes	4 (20)	0 (0)	0 (0)	0 (0)	4 (9.1)	0.251
No	16 (80)	9 (100)	8 (100)	7 (100)	40 (90.9)	
History of alcohol (*n* = 44)						
Yes	0 (0)	1 (11.1)	0 (0)	0 (0)	1 (2.3)	0.545
No	20 (100)	8 (88.9)	8 (100)	7 (100)	43 (97.7)	
Location of tumor						
Oral cavity	38 (71.7)	19 (70.4)	12 (63.2)	10 (62.5)	79 (68.7)	0.814
Lip	1 (1.9)	1 (3.7)	0 (0)	1 (6.3)	3 (2.6)	
Tongue	11 (20.8)	6 (22.2)	7 (36.8)	5 (31.3)	29 (25.2)	
Soft palate	3 (5.7)	1 (3.7)	0 (0)	0 (0)	4 (3.5)	
Tumor stage						
T1	12 (22.6)	7 (25.9)	9 (47.4)	3 (18.8)	31 (27)	0.003
T2	33 (62.3)	13 (48.1)	5 (26.3)	3 (18.8)	54 (47)	
T3/T4	8 (15.1)	7 (25.9)	5 (26.3)	10 (62.5)	30 (26.1)	
Tumor depth						
< 2 cm	46 (86.8)	22 (81.5)	18 (94.7)	12 (75)	98 (85.2)	0.377
≥ 2 cm	7 (13.2)	5 (18.5)	1 (5.3)	4 (25)	17 (14.8)	
Nodal stage						
No	30 (56.6)	14 (51.9)	13 (68.4)	6 (37.5)	63 (54.8)	0.433
N1	10 (18.9)	3 (11.1)	1 (5.3)	3 (18.8)	17 (14.8)	
N2a	0(0)	0 (0)	0 (0)	0 (0)	0 (0)	
N2b	12 (22.6)	8 (29.6)	5 (26.3)	6 (37.5)	31 (27)	
N2c	0 (0)	2 (7.4)	0 (0)	1 (6.3)	3 (2.6)	
N3	1 (1.9)	0 (0)	0 (0)	0 (0)	1 (0.9)	
Extranodal extention						
Not present	43 (81.1)	19 (70.4)	14 (73.7)	9 (56.3)	85 (73.9)	0.236
Present	10 (18.9)	8 (29.6)	5 (26.3)	7 (43.8)	30 (26.1)	
Histological subtypes						
Non-keratinizing	8 (15.1)	3 (11.1)	3 (15.8)	3 (18.8)	17 (14.8)	0.823
Keratinizing	33 (62.3)	15 (55.6)	10 (52.6)	7 (43.8)	65 (56.5)	
Non-keratinizing with maturation	12 (22.6)	9 (33.3)	6 (31.6)	6 (37.5)	33 (28.7)	
Histologic grade						
Grade-I	19 (35.8)	7 (25.9)	4 (21.1)	1 (6.3)	31 (27)	0.281
Grade-II	27 (50.9)	18 (66.7)	13 (68.4)	13 (81.3)	71 (61.7)	
Grade-III	7 (13.2)	2 (7.4)	2 (10.5)	2 (12.5)	13 (11.3)	
Lymphovascular invasion						
Not present	52 (98.1)	27 (100)	19 (100)	16 (100)	114 (99.1)	1.000
Present	1 (1.9)	0 (0)	0 (0)	0 (0)	1 (0.9)	
Perineural invasion						
Not present	48 (90.6)	23 (85.2)	15 (78.9)	13 (81.3)	99 (86.1)	0.493
Present	5 (9.4)	4 (14.8)	4 (21.1)	3 (18.8)	16 (13.9)	
Radiation (*n* = 45)						
Yes	12 (60)	7 (70)	4 (50)	2 (28.6)	25 (55.6)	0.366
No	8 (40)	3 (30)	4 (50)	5 (71.4)	20 (44.4)	
Chemotherapy (*n* = 45)						
Yes	12 (60)	6 (60)	4 (50)	2 (28.6)	24 (53.3)	0.513
No	8 (40)	4 (40)	4 (50)	5 (71.4)	21 (46.7)	

Chi-square test was applied

*P* value ≤ 0.05 considered as significant

## Discussion

Immunohistochemical staining of 115 carcinomas of the squamous cell origin from the head and neck biopsies was carried out to find out the frequency of EGFR expression in head and neck SCC of our population and to determine an association of EGFR over-expression with unfavorable prognostic features including advanced tumor grade, tumor size, nodal metastasis, and recurrence status in our population. 45.2% of our cases revealed EGFR over-expression, and significant association of EGFR was noted with tumor stage and disease-free survival, which are among the most important prognostic factors in head and neck SCC.

High expression of EGFR in head and neck SSC has been reported by previous studies [7]. In a similar study conducted by Sarkis et al., the EGFR immunostaining was positive in 87.5% of the cases [8]. Likewise, a high expression of EGFR 73.42% was found in another study conducted by Laimer et. al. Moreover, 92.3% of cases were positive for EGFR staining in the study conducted by Hiraishi et.al [13]. As a consequence of inconsistency in methods of evaluation of EGFR, incongruity exists between studies reporting EGFR as a prognostic marker of squamous cell carcinoma. Over-expression of EGFR correlates with aggressive tumor behavior and decreased life expectancy [14]. In our study, we found that 45.2% of cases of SCC of head and neck showed positive EGFR staining, and small numbers of cases (8.7%) were focal positive, whereas 46.1% of cases showed negative EGFR staining. Comparison with other studies reveals that EGFR expression is relatively low in our population compared the reported data.

In an antecedent qualitative literature review conducted by Piccirillo et al., no significant association between age, gender, tumor location, grade and lymph node involvement, and prognosis of the disease was expressed [15]; however, these tumor characteristics play a meaningful role in disease management. Similarly, Grandis et al. expressed that no significant association was found between these clinico-pathologic characteristics of tumor with regard to meaningful clinical outcomes [16]. Hence, these characteristics are regarded as inefficient predictors of disease outcome, but frequently considered in designing personalized therapeutic regimes. In congruence with these previous studies, we found no significant association between EGFR over-expression with many clinico-pathologic characteristics including age, gender, tumor depth, nodal stage, and histological stage in our study; however, significant association was noted with tumor stage. Hiraishi et al. in a study involving 52 cases of oral SCC revealed significant association of EGFR over-expression with tumor invasion; however, association with other prognostic parameters was not found.

Another role of IHC expression of EGFR resides in its ability to differentiate between healthy/hyperplastic and diseased/dysplastic (premalignant) proliferative changes, as many authors found a significantly high expression of EGFR in premalignant squamous mucosa compared to hyperplastic/non-premalignant squamous epithelium [17, 18]. Similar to our study, Srivastava et al. also did not found a significant association of various risk factors with EGFR expression [19]. In contrast to our study, Panday et al., in a study involving 24 locally advanced HNSCC cases, did not found any significant association of EGFR expression with disease-free survival in patients taking neoadjuvant chemotherapy [20]. Similarly, Kumar et al. suggested that EGFR expression did not predict response to neoadjuvant chemotherapy [21]. On the other hand, adjuvant anti-EGFR therapy (e.g., Cetuximab) significantly improves survival in patients with advanced HNSCC [22]. Zafar et al. did not found any significant association of EGFR with tumor grade; however, association with other prognostic factors was not tested in their study [23].

We did not perform molecular studies in our cases (limitations of the study) because, for the use of anti-EGFR-targeted therapy, it is widely accepted that only IHC EGFR expression is not enough for patient selection that may benefit from EGFR-directed therapy. The reason behind that is IHC EGFR expression does not necessarily correlate with underlying gene amplification. Bermardes et al. reported no significant association of EGFR over-expression with gene amplification by FISH or CISH. In their study, although IHC over-expression of EGFR was noted in 53.8% of cases, gene amplification was seen in only 5.8 and 15.4% by CISH and FISH respectively [24].

## Conclusion

We found a significant association of EGFR expression with tumor stage and disease-free survival, which are the most important prognostic factors in head and neck squamous cell carcinoma; therefore, EGFR expression can be used as a prognostic biomarker in head and neck squamous cell carcinoma. On the other hand, we suggest that molecular studies should be performed in squamous cell carcinoma of head and neck in our setup to identify patients that can benefit from anti-EGFR therapy.
